# A Rare Case of Laryngeal Non-IgM Lymphoplasmacytic Lymphoma

**DOI:** 10.7759/cureus.29136

**Published:** 2022-09-13

**Authors:** Michelle K Hong, Albert Y Han, Travis L Shiba

**Affiliations:** 1 Head and Neck Surgery, University of California Los Angeles David Geffen School of Medicine, Los Angeles, USA; 2 Otolaryngology, MD Anderson Cancer Center, Houston, USA

**Keywords:** laryngeal mass, non-hodgkin's lymphoma, non-igm, lymphoplasmacytic, laryngeal lymphoma

## Abstract

Laryngeal lymphoplasmacytic lymphoma has been previously reported only a handful of times in the literature. It can be difficult to diagnose without significant histologic workup and proper methodology. Here, we demonstrate the first known case of laryngeal lymphoplasmacytic lymphoma with non-immunoglobulin M (IgM) features. In this case report, a 79-year-old female with seropositive rheumatoid arthritis presented with five months of dysphonia and dyspnea on exertion. Lab studies revealed high levels of serum IgA and IgG. Flexible laryngoscopy and computed tomography of the neck showed a left supraglottic submucosal mass, which was surgically excised with a carbon dioxide laser. The histology of the mass confirmed the diagnosis of lymphoplasmacytic lymphoma. The patient was treated with 30.6 Gy of radiation therapy and eight cycles of rituximab with successful remission of her lymphoma and no evidence of disease recurrence six months after treatment completion. Lymphoplasmacytic lymphoma without corresponding IgM gammopathy is unusual and has been shown to have a higher frequency of extramedullary involvement. This is the first known manifestation of non-IgM lymphoplasmacytic lymphoma in the larynx.

## Introduction

Laryngeal lymphoma is a rare presentation of lymphoma, with less than 100 cases reported in the literature to date [1]. Specifically, laryngeal lymphoplasmacytic lymphoma (LPL) is extremely rare and has its own unique considerations in diagnostic workup and treatment. This patient demonstrates the characteristics of a non-immunoglobulin M (IgM) LPL, which, to our knowledge, has never been documented as a laryngeal primary lesion in prior literature.

Laryngeal LPL can be difficult to diagnose due to its nonspecific symptoms and indolent nature [2]. The disease often presents with progressive symptoms, including dyspnea, dysphagia, and dysphonia [3]. Additionally, it is not often accompanied by the typical systemic symptoms associated with other lymphomas, known as B-symptoms [2]. Diagnosis is often established with a thorough immunohistochemical workup after surgical excision or biopsy. Treatment of laryngeal LPL often involves a combination of surgery, localized radiation, and systemic chemotherapy [3,4]. Here, we present the first known report of a patient with non-IgM laryngeal LPL with intraoperative endoscopic images and a review of the literature.

## Case presentation

**Figure 1:**
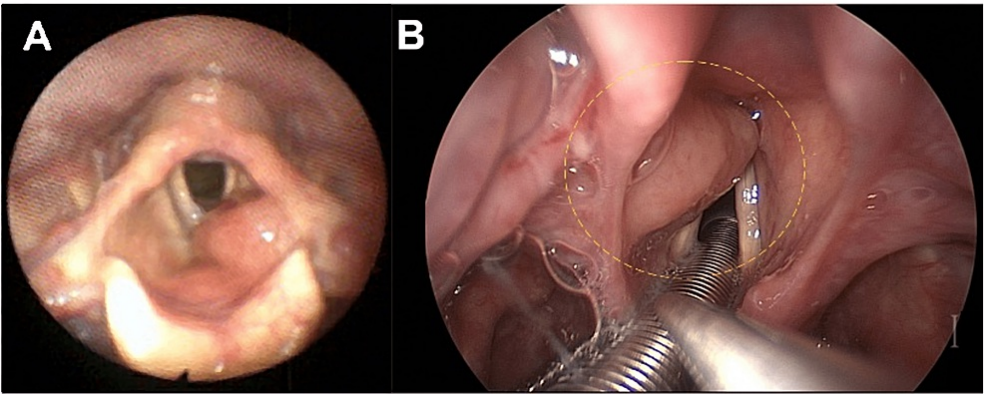
Endoscopic images of the left supraglottic mass Flexible laryngoscopy (Panel A) in the clinic demonstrated a supraglottic submucosal mass that obstructs approximately 50% of the laryngeal inlet when relaxed. This mass is again visualized in the operating room using a high-definition camera during surgical biopsy and resection (Panel B). The yellow dotted circle indicates the location of the submucosal supraglottic mass.

**Figure 2:**
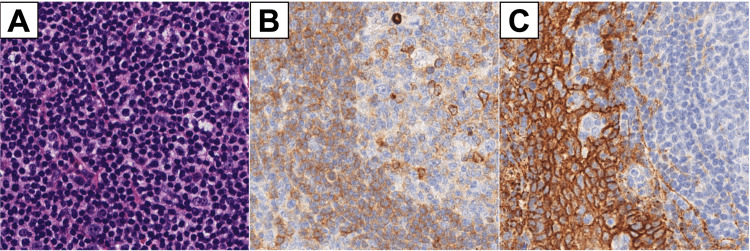
Immunohistochemical analysis of the excised supraglottic mass Panel A: Hematoxylin and eosin stain revealing sheets of small lymphoid cells with large confluent aggregates of plasma cells. Panel B: CD138 immunohistochemical stain using horseradish peroxidase; brown color indicates areas of positive staining in large aggregates of plasma cells. Panel C: IgM immunohistochemical stain; brown color indicates areas of strong positive cytoplasmic staining in plasma cells.

A 79-year-old female with a history of hypertension, chronic sinusitis, asthma, and seropositive deforming rheumatoid arthritis with loss of cervical lordosis (antinuclear antibody (ANA) titer 1:1280) presented to the clinic with five months of dysphonia, dyspnea on exertion, excessive throat mucus, and throat pain. The patient denied fevers, chills, night sweats, neck masses, or unintentional weight loss. On physical exam, there was no cervical lymphadenopathy and no change in neck mobility from baseline. There was no hepatosplenomegaly. The patient had a harsh and rough voice quality with a moderately restricted pitch range. Flexible laryngoscopy showed a left-sided supraglottic submucosal mass without extension to the true vocal folds obstructing 50% of the laryngeal inlet (Figure 1). She had normal vocal cord mobility bilaterally. A computed tomography (CT) scan of the neck with contrast showed a 1.4 x 1.7 x 4 cm supraglottic soft tissue mass to the left of the pre-epiglottic space and left aryepiglottic fold thickening, resulting in moderate airway narrowing. There was no cervical lymphadenopathy identified on the CT scan. Laboratory studies demonstrated increased IgG at 1894 mg/dL and increased IgA at 754 mg/dL but normal IgM at 147 mg/dL. Kappa free light chains were elevated at 82.5 mg/L, lambda free light chains were elevated at 48.1 mg/L, and the kappa/lambda ratio was elevated at 1.72. An endoscopic partial laryngectomy with a carbon dioxide laser and biopsy forceps was performed to remove the mass. Immunohistochemical staining of the excised mass revealed sheets of small lymphoid cells with large confluent aggregates of plasma cells with Dutcher bodies (Figure 2A) that expressed CD138 (Figure 2B) and IgM (Figure 2C). Flow cytometry results were consistent with a monotypic, kappa-restricted B-cell population negative for CD5, CD10, and CD23. Histology and flow cytometry results were consistent with laryngeal LPL.

After surgical excision, this patient completed radiation therapy of 30.6 Gy to the glottis and supraglottic area in 17 fractions. She subsequently received eight cycles of rituximab. This treatment regimen resulted in the full resolution of the patient’s dysphonia. A positron emission tomography (PET) scan after treatment completion showed no evidence of disease recurrence, indicating a complete response. Flexible laryngoscopy six months after treatment completion showed no visible mass in the left supraglottic region.

## Discussion

The differential diagnosis of a laryngeal mass is broad and ranges from neoplasms, including laryngeal lymphoma, to non-cancerous lesions, including laryngocele, vocal cord polyps, and sarcoidosis [5]. Laryngeal lymphoma is rare in this differential, only constituting less than 1% of all laryngeal neoplasms [6]. The LPL subtype is even more uncommon, with only three prior reports of it in the literature involving the larynx specifically [7-9]. Patients often present with non-specific symptoms of the larynx, including dysphonia, dysphagia, dyspnea, and globus sensation [2]. Patients may describe dyspnea in a more advanced disease [2]. Laryngeal lymphoma most commonly presents in the supraglottis due to lymphoid tissue in the ventricle and lamina propria of the mucosa in this region [9]. The gross appearance of this mass on laryngoscopy was consistent with other reports of laryngeal lymphoma, typically presenting as a smooth submucosal swelling [6]. As demonstrated in this patient, there is also a propensity for a laryngeal lymphoma to arise on the left side for unclear reasons [10]. While patients with NHL may present with systemic B-symptoms such as fevers, chills, night sweats, and unintentional weight loss, prior reports of laryngeal lymphoma presentation do not typically describe B-symptoms [2,8,9]. Thus, the presence or absence of B-symptoms is not reliable for the diagnosis of laryngeal lymphoma. A laryngeal lymphoma should be considered in the differential diagnosis of submucosal masses in the supraglottis with an indolent onset in late adulthood.

LPL is a rare, low-grade NHL with an incidence of 1000-1500 new cases per year in the United States, and most patients survive seven to eight years after diagnosis [3]. LPL commonly presents in conjunction with other autoimmune diseases, such as Sjogren’s syndrome and rheumatoid arthritis, the latter of which was seen in this patient [9]. While NHL originating in extranodal sites is uncommon, ranging from 10-35%, it is more common in LPL compared to other NHLs [2,9]. However, the larynx is a rare site of extramedullary NHL localization, with fewer than 100 cases of laryngeal lymphoma published in the literature, and only three other reports describing LPL specifically involving the larynx [1,7-9].

LPL is typically a diagnosis of exclusion within NHLs, as other forms of small B-cell lymphoma should be eliminated prior to diagnosing LPL [3]. Histology and flow cytometry in this patient were instrumental in establishing the diagnosis in this patient. Mantle cell lymphoma is CD5+, follicular lymphoma is CD10+, and cutaneous T-cell lymphoma is CD20- [5]. Histology from this patient eliminates these other lymphoma subtypes from contention. Additionally, CD138 has been shown to help distinguish LPL from marginal zone lymphoma, with 47% of patients with LPL demonstrating moderate to high expression of CD138 compared to low expression of CD138 in marginal zone lymphoma patients [11]. The patient was also tested for the presence of the MDY88 L265P mutation, as up to 90% of LPL patients express this mutation [3]. The patient tested negative for the mutation; while this is uncommon, given the nonspecific nature of the MDY88 L265P mutation, the presence of the mutation is not required for a diagnosis of LPL [3].

LPL is typically associated with IgM monoclonal gammopathy, and when both are present, this is also known as Waldenstrom macroglobulinemia (WM) [3,12]. The patient had incidentally been found to have hypergammaglobulinemia prior to the presentation of dysphonia, and further evaluation of this gammopathy demonstrated an increase in IgG and IgA with normal IgM. This was initially attributed to her existing rheumatoid arthritis (RA) or the result of the treatments she was on for RA leading to abnormalities in her immunoglobulin levels. However, in retrospect, this could have been the first indication of her lymphoma. Non-IgM LPL is rare within LPL and has been shown to demonstrate extramedullary involvement much more frequently than in WM patients, as seen in this patient’s laryngeal presentation [13]. Non-IgM LPL also has a lower frequency of the MDY88 mutation when compared to WM patients, which can also explain the lack of the mutation in our patient [13]. Although this patient’s excised lymphoma demonstrated IgM staining, IgG and IgA expression could not be excluded due to a high background on the staining procedure. Additionally, her serum IgM levels remaining normal make this a very unusual presentation of LPL that demonstrates properties more akin to a non-IgM LPL. As a result, this is the first presentation, to our knowledge, of a primary laryngeal non-IgM LPL. This also demonstrates the importance of following up on laboratory abnormalities that could have been the early signs of her LPL but were at first attributed to her RA or its treatments.

The treatment for primary laryngeal lymphomas includes radiation therapy and chemotherapy. The tendency for extranodal NHL to stay localized without systemic spread makes this disease more responsive to local radiation therapy; the use of 30-50 Gy has been shown to achieve disease control in prior literature [2,6]. Laryngeal lymphomas have typically been shown to have a good prognosis with surgery, radiation, and chemotherapy [8]. Additionally, patients with non-IgM LPL have previously been treated with rituximab-based therapies with success [13]. This patient received 30.6 Gy of radiation and rituximab after surgical excision and achieved complete remission of her lymphoma. This highlights the importance of prompt multidisciplinary treatment upon diagnosis and treatment of this disease.

## Conclusions

Laryngeal lymphoma is a rare disease that is often overlooked as a potential diagnosis in the broad differential for a laryngeal mass. This patient demonstrated an unusual presentation of a non-IgM laryngeal LPL without systemic symptoms and without the serum IgM gammopathy typical of LPL, which has not been seen before in the literature. She was treated with an interdisciplinary approach comprising surgery, radiation, and systemic chemotherapy. This case highlights the unique presentation of this rare lymphoma and demonstrates the need to consider this diagnosis primarily with supraglottic and submucosal laryngeal lesions even in the absence of the typical B-symptoms of lymphoma.
